# High‐Density SNP Genotyping Reveals High Population Connectivity and Limited Spatial Genetic Structure in *Apodemus flavicollis* and *Apodemus sylvaticus*

**DOI:** 10.1002/ece3.74345

**Published:** 2026-09-08

**Authors:** Maria Chiara Fabbri, Matilde Martini, Giovanna Donati, Giuseppe Coiro, Giuseppe Luzzi, Luciano Ferraro, Donata Coppola, Riccardo Bozzi

**Affiliations:** ^1^ Department of Agriculture, Food, Environment, and Forestry (DAGRI) University of Florence Florence Italy; ^2^ National Park Authority of the Lucanian Apennines – Val d'Agri – Lagonegrese Potenza Italy

**Keywords:** *Apodemus* species, cross‐species genotyping, forest fragmentation, genetic diversity, population genetic structure, SNP array

## Abstract

High‐density SNP arrays are increasingly used in ecological and evolutionary studies, yet their application in wild species remains challenging. In this study, we evaluated the performance of the Affymetrix Axiom Mouse HD array, originally developed for *Mus musculus* , in two wild small mammals, *Apodemus flavicollis* and *Apodemus sylvaticus* , with particular focus on genetic diversity and population connectivity across seven sampling sites within a fragmented landscape. A total of 96 individuals (43 *A. flavicollis* and 53 *A. sylvaticus* ) were genotyped using a 616K SNP array. After quality control filtering for missingness and minor allele frequency, more than 160,000 high‐quality autosomal SNPs were retained for each species. Despite being designed for a different species, the array effectively discriminated between *A. flavicollis* and *A. sylvaticus* , with principal component analysis clearly separating the two species. Levels of genetic diversity were comparable across sites, with mean observed heterozygosity around 0.33 and consistently negative *F*_IS_ values, indicating a slight excess of heterozygotes. Population structure analyses revealed extremely weak spatial genetic differentiation. ADMIXTURE supported a single genetic cluster (*K* = 1) within each species, while analysis of molecular variance attributed more than 99% of genetic variation to within‐individual components. Pairwise relationship analyses showed that related individuals were not confined to single sites but occurred across sampling locations, supporting ongoing gene flow even across the fragmented landscape. No significant isolation‐by‐distance pattern was detected. Overall, our results indicate high population connectivity and limited spatial genetic structuring in both species across the study area, consistent with the documented dispersal capacity of these species at the spatial scale investigated. Moreover, this study demonstrates that high‐density SNP arrays can provide powerful genomic tools for investigating dispersal dynamics and population structure in closely related wildlife species under habitat fragmentation, where subtle genetic patterns may otherwise remain undetected.

## Graphical abstract

We evaluated the performance of a high‐density cross‐species SNP array to discriminate and genetically characterise *Apodemus flavicollis* and *Apodemus sylvaticus* in a fragmented agriforest landscape. The SNP data clearly separated the two species and revealed high genetic diversity, weak spatial genetic structure and no isolation‐by‐distance within each species. These results indicate high population connectivity despite habitat fragmentation, highlighting the utility of SNP arrays for landscape and population genetic studies in non‐model species.

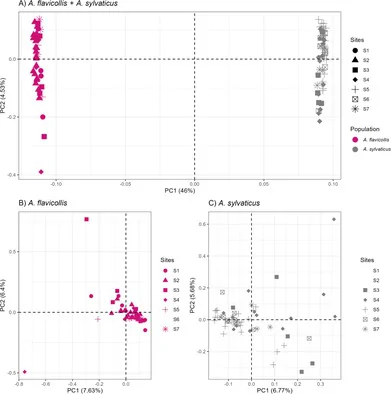

## 1 Introduction

Small and isolated populations may undergo a progressive erosion of genetic diversity (Clough et al. 2026). Reduced genetic variability is associated with declines in population fitness (Kardos et al. 2021), affecting key parameters such as population growth rate, demographic resilience and long‐term adaptive capacity (Leberg 1993). In such populations, increased inbreeding and the effect of genetic drift can further reduce heterozygosity and increase the risk of local extinction (Lacy 1997; Kardos et al. 2021).

Species extinction is often the result of multiple human‐induced threats acting simultaneously (Frankham et al. 2014). Among these, habitat fragmentation is widely recognised as a major driver of reduced connectivity among populations, limiting gene flow and exacerbating the effects of genetic drift and inbreeding (DiBattista 2008; Stevens et al. 2018). The ecological consequences of habitat fragmentation have been extensively investigated across a wide range of taxonomic groups, including plants, invertebrates, birds and mammals (Haddad et al. 2015). However, habitat fragmentation also affects genetic variation in natural populations, with important consequences for population genetic structure (DiBattista 2008). Habitat fragmentation, through habitat loss and reduced connectivity, can disrupt gene flow, increase genetic isolation among populations and ultimately accelerate the loss of genetic diversity (Frankham et al. 2002). Integrating genetic data with ecological and demographic approaches is therefore essential for achieving a more comprehensive understanding of these processes, particularly when supported by robust, carefully designed genetic tools capable of detecting subtle changes in population connectivity and genetic structure.

Among mammals, small rodents have been used as model organisms for investigating the genetic consequences of habitat fragmentation (Gaines et al. 1997; Lino et al. 2019), due to their relatively short generation times, their distribution in patchy populations with rapid turnover, selective habitat use and their strong sensitivity to landscape characteristics (Mortelliti et al. 2009). Among small rodents, the wood mouse *Apodemus sylvaticus* (Linnaeus, 1758) and the yellow‐necked mouse *Apodemus flavicollis* (Melchior, 1834) are two closely related and widely distributed species across Europe, often occurring in sympatry (Bartolommei et al. 2016). Both species are generally associated with woodland habitats. However, *A. sylvaticus* exhibits greater ecological flexibility and can occupy a wide range of habitats, whereas *A. flavicollis* is more closely associated with forest environments (Amori et al. 2008). Despite their ecological relevance, identifying these species based on external morphology can be challenging, particularly in areas of sympatry where morphological and morphometric traits largely overlap (Niethammer 1978; Filippucci et al. 1984). Misidentification may therefore introduce biases in population genetics, especially when species‐specific responses to environmental factors are investigated.

Until recently, traditional population genetic and landscape genetic studies of mammal dispersal, gene flow and/or spatial genetic structure have largely relied on microsatellite markers (Storfer et al. 2010; Bani et al. 2017). These markers have historically been widely used due to their high polymorphism, relative ease of development and relatively low genotyping costs (Selkoe and Toonen 2006; Guichoux et al. 2011). However, microsatellites also present several limitations, including issues related to scoring reproducibility, difficulties in standardising datasets across laboratories and the relatively limited number of loci typically analysed (McMahon et al. 2014). In contrast, high‐throughput genotyping approaches based on single‐nucleotide polymorphisms (SNPs) provide a much larger number of markers distributed across the genome, enabling more accurate estimates of genetic diversity, population structure and demographic history, resolving subtle genetic structuring not detected by microsatellites (von Thaden et al. 2020; Skey et al. 2023). Consequently, SNP‐based approaches have progressively replaced microsatellites in many fields, particularly in livestock and companion animal genetics (von Thaden et al. 2017). Despite these advantages, the application of SNP‐based methods in wild species remains comparatively limited (McMahon et al. 2014). One of the main constraints is the cost of developing and implementing genotyping platforms. SNP arrays typically rely on reference genome assemblies to identify and select polymorphic sites. Therefore, the availability and quality of a reference genome are fundamental prerequisites that are not expected in wildlife species (von Thaden et al. 2020). Ideally, a species‐specific SNP chip should be developed for each species in order to maximise genotyping efficiency and genomic coverage. However, the development of species‐specific arrays is economically demanding and therefore rarely feasible for most non‐model organisms, as it is driven by needs in fields such as human genetics and plant and animal breeding (McMahon et al. 2014).

For many non‐model species, including those of the genus *Apodemus*, the application of SNP arrays remains largely unexplored. In particular, their performance has not yet been evaluated in the species investigated in the present study. Therefore, the primary objective of this work was to test the performance of a non‐species‐specific SNP array in discriminating and genetically characterising *A. sylvaticus* and *A. flavicollis* . This is particularly relevant for closely related, morphologically similar species such as *A. sylvaticus* and *A. flavicollis*. In addition, as a secondary objective, we explored whether the SNP‐based approach could detect patterns of genetic diversity and inbreeding within populations potentially associated with population isolation in the study system. We further characterised population genetic structure and relatedness among populations to indirectly infer patterns of functional connectivity and underlying levels of gene flow. Together, these analyses provided proof of concept for the application of this genomic approach in future population genetic studies of non‐model species in fragmented landscapes.

## 2 Materials and Methods

### 2.1 Study Population and Sampling

**FIGURE 1:**
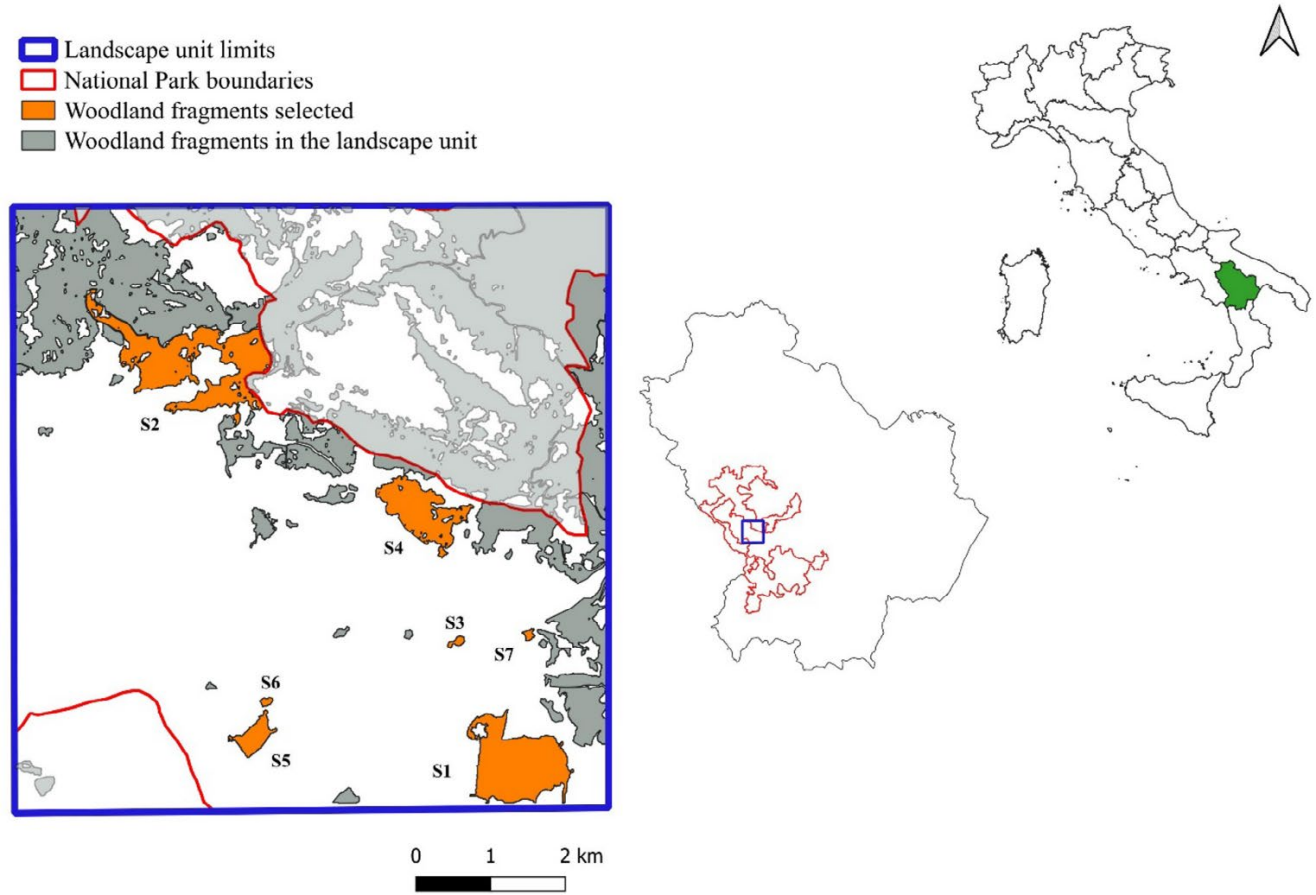
Study area and the fragmented landscape unit (64 km^2^) selected to investigate the application of SNP arrays for species discrimination, genetic characterisation, assessment of genetic diversity and inbreeding patterns and analysis of population genetic structure in *A. sylvaticus* and *A. flavicollis* . Orange polygons indicate the sampled woodland fragments (*n* = 7), all located outside the boundaries of the protected area.

The study area is located in the upper part of the Agri Valley, in the Province of Potenza (southern Italy), within the Basilicata region. It includes the Appennino Lucano – Val d'Agri – Lagonegrese National Park and the surrounding agroforestry landscape bordering the protected area (Figure 1).

The selection of sampled forest fragments was based on data collected in 2022 using indirect sampling methods, which allowed the identification of the presence of the genus *Apodemus* in fragments located in the agroforestry matrix outside the boundaries of the protected area. A total area of 64 km^2^ was defined as the landscape unit, predominantly composed of deciduous oak woodlands ( *Quercus cerris* and *Quercus pubescens*). However, only forest fragments located outside the protected area were considered for sampling. Within this landscape unit, selected fragments represented a gradient of isolation, calculated as the minimum distance to the nearest forest patch, ranging from 10 m to 494.77 m. Within each fragment, a grid of 36 Sherman live traps (5.2 × 6.4 × 16.5 cm; Sherman Traps, Tallahassee, Florida, USA) was established, with traps spaced 10 m apart and activated for three consecutive nights. Traps were baited with a mixture of apple, peanut butter and crushed hazelnuts. Sampling was conducted from May to November 2025, across two seasonal sessions (spring/summer and autumn). All captured individuals were marked via subcutaneous implantation of a PIT tag (7 × 1.4 mm; Passive Integrated Transponder, ISO 11784) to assign a unique identification code. Each individual was weighed, sexed and additional biometric measurements were recorded. Individuals were preliminarily assigned to *A. flavicollis* or *A. sylvaticus* based on external morphological and biometric characteristics, following the identification criteria (Amori et al. 2008; Bartolommei et al. 2016; Paolucci and Bon 2022), while accounting for the known morphological overlap between the two species. Tissue samples were collected from the outer ear using sterilised scissors. Samples were stored in tubes containing 95% ethanol and kept at −20°C until transport to the laboratory and subsequent analysis.

### 2.2 SNP Genotyping

All individuals were genotyped using the Affymetrix Axiom MouseHD (Thermo Fisher Scientific), a high‐density SNP genotyping platform derived from the Mouse Diversity Genotyping Array (https://documents.thermofisher.com/TFS‐Assets/LSG/manuals/MAN0017713_702887_MouseDiversityArray_PI.pdf) originally developed for laboratory mouse strains. The array was designed to maximise genetic diversity coverage and discrimination among murine lineages by incorporating SNPs from multiple sources, including NIEHS/Perlegen datasets, the Ken Paigen laboratory resources, B6 and MSM strain singletons, wild‐derived strains, sex chromosomes (X and Y) and mitochondrial loci. SNPs included in the original Mouse Diversity Genotyping Array were selected based on their performance across 84 distinct samples screened by The Jackson Laboratory and subsequently validated using 148 samples comprising classical inbred strains, F1 crosses among classical strains and crosses involving wild‐derived strains. Although originally developed for *Mus musculus* , this genotyping platform can be justified for studies of the genus *Apodemus* given their relatively close phylogenetic relationship within Murinae. Phylogenetic analyses based on mitochondrial markers and the nuclear IRBP gene have shown that *Apodemus* is more closely related to *Mus* than to other murine genera such as *Rattus* or *Micromys*, supporting their status as sister taxa (Michaux et al. 2002; Çetintürk et al. 2025). Divergence time estimates place the split between *Mus* and *Apodemus* at approximately 9–10 million years ago, representing a relatively recent divergence on the genomic timescale. Based on these assumptions, a substantial proportion of loci designed for *Mus musculus* is expected to be conserved and informative in *Apodemus*, supporting the suitability of the Axiom MouseHD array tool for investigating population genetic structure in this genus. Both species of *Apodemus* considered in this study possess a diploid chromosome number of 2*n* = 48 (Topashka‐Ancheva and Yordanova 2008). However, because the Affymetrix Axiom MouseHD array was originally designed for *Mus musculus* , SNP markers are annotated and mapped according to the *Mus musculus* reference genome, which comprises 19 autosomal chromosome pairs. To characterise the SNP chip features and their performance in our species, we calculated missingness, allele frequencies and marker distribution across chromosomes using the—missing and—freq flags, respectively, in PLINK software (Chang et al. 2015). Quality control (QC) was carried out on the entire dataset ( *A. sylvaticus* and *A. flavicollis* ) and on each species separately. Missing genotypes and individuals with a missingness threshold greater than the empirically chosen threshold were removed, using the—geno and—mind flags in PLINK v1.9 (Chang et al. 2015). The minor allele frequency (MAF) threshold was applied using the—maf flag in PLINK v1.9. Only autosomal SNPs were retained. Linkage disequilibrium pruning was not applied to maximise the power for identifying weak genetic differentiation, particularly because the physical distribution and linkage of retained SNPs in the target species are not known with the same confidence as in the reference species (Bercovich et al. 2025).

### 2.3 Genetic Diversity

Genetic diversity was assessed using the R package *dartR* (Mijangos et al. 2022). Observed heterozygosity (*H*_o_), expected heterozygosity (*H*_e_), unbiased expected heterozygosity (u*H*_e_) and the inbreeding coefficient (*F*_IS_) were calculated using the *gl.report.heterozygosity* function. The estimates were calculated as follows:

$$\text{Observed heterozygosity}\;\left(H_{o}\right)=\text{number of heterozygotes}/n\_\mathrm{Ind}$$

where *n_Ind* is the number of individuals without missing data.

$$\text{Expected heterozygosity}\;\left(H_{e}\right)=1-\left(p^{2}+q^{2}\right)$$

where *p* is the frequency of the reference allele and *q* is the frequency of the alternative allele.

$$\begin{array}{c} \text{Unbiased expected heterozygosity}\;\left(uH_{e}\right)\\ \;=H_{e}*\left(2*n\_\mathrm{Ind}/\left(2*n\_\mathrm{Ind}-1\right)\right) \end{array}$$

$$\;\text{Inbreeding coefficient}\;\left(F_{\text{IS}}\right)=1-\left(\text{mean}\;\left(H_{o}\right)/\text{mean}\;\left(uH_{e}\right)\right)$$

Differences in heterozygosity among sites were tested using the *gl.test.heterozygosity* function with 999 permutations.

### 2.4 Population Structure

The performance of the SNP chip in non‐conventional species was assessed by testing its ability to discriminate and genetically characterise *A. sylvaticus* and *A. flavicollis* . Specifically, we evaluated whether individuals could be reliably resolved into distinct genetic clusters. To this end, the population genetic structure of the two wild species was characterised using two different approaches: Principal Components Analysis (PCA) and Admixture analysis. PCA was performed using the—pca flag in PLINK v1.9 (Chang et al. 2015), which produces orthogonal projections of the original genotype data. Two PCAs were conducted: the first on the entire dataset, including both *A. sylvaticus* and *A. flavicollis*, to assess the discriminant power of the SNP chip; the second separately for each species to quantify within‐species genetic structure.

Admixture analyses were performed using the R package *LEA* (Frichot and François 2015). Analyses were first conducted on the complete dataset and then separately for each species, considering sampling sites as putative ancestral populations. The number of ancestral populations (*K*) was inferred using the cross‐entropy criterion. For each fixed value of *K*, the run with the lowest cross‐entropy was retained as the best‐supported solution. For the complete dataset, 10 replicate runs were performed, testing *K* = 1–3. For *A. flavicollis* , 10 replicates were conducted for *K* values ranging from 1 to 6, corresponding to the number of sampling sites. For *A. sylvaticus*, 10 replicates were performed for *K* = 1–5.

To further evaluate genetic relatedness among individuals, pairwise genomic relationship coefficients were calculated within each species using the –make‐rel flag in PLINK (Chang et al. 2015). This approach estimates the genomic relationship matrix (GRM) according to Yang et al. (2011), where coefficients quantify the proportion of the genome shared identical‐by‐state (IBS) based on the population allele frequencies. Coefficients close to zero indicate unrelated individuals, positive values indicate higher genomic similarity, whereas negative values indicate lower‐than‐average genomic similarity. The resulting matrices were visualised as heatmaps using the R package *pheatmap* (Kolde 2025), where individuals are grouped according to sampling site. Mean and standard deviation of relatedness were also calculated at both species and site levels.

Analysis of Molecular Variance (AMOVA) was conducted using the R package *poppr* (v.2.9.8) (Kamvar et al. 2014), partitioning genetic variation among populations (here, sites), among individuals within sites and within individuals. Statistical significance was assessed using permutation tests (*n* = 999). Finally, a Mantel test was performed to assess the correlation between genetic and geographic distances among sampling sites, providing insight into patterns of isolation by distance (permutations = 999).

All results were visualised using the R package *ggplot2* (Wickham 2016). These complementary analyses were used to characterise population genetic structure and to evaluate patterns consistent with genetic differentiation and connectivity among sites.

## 3 Results

### 3.1 Study Population and Sampling

A total of 104 individuals were captured in the selected forest fragments. We found *Apodemus* individuals in all the sampled forest fragments. The mean number of individuals captured per site was 15 ± 2, ranging from 8 to 23. The number of animals in the forest fragments followed the isolation gradient, with the most isolated forest patches hosting the fewest individuals.

### 3.2 SNP Genotyping

**FIGURE 2:**
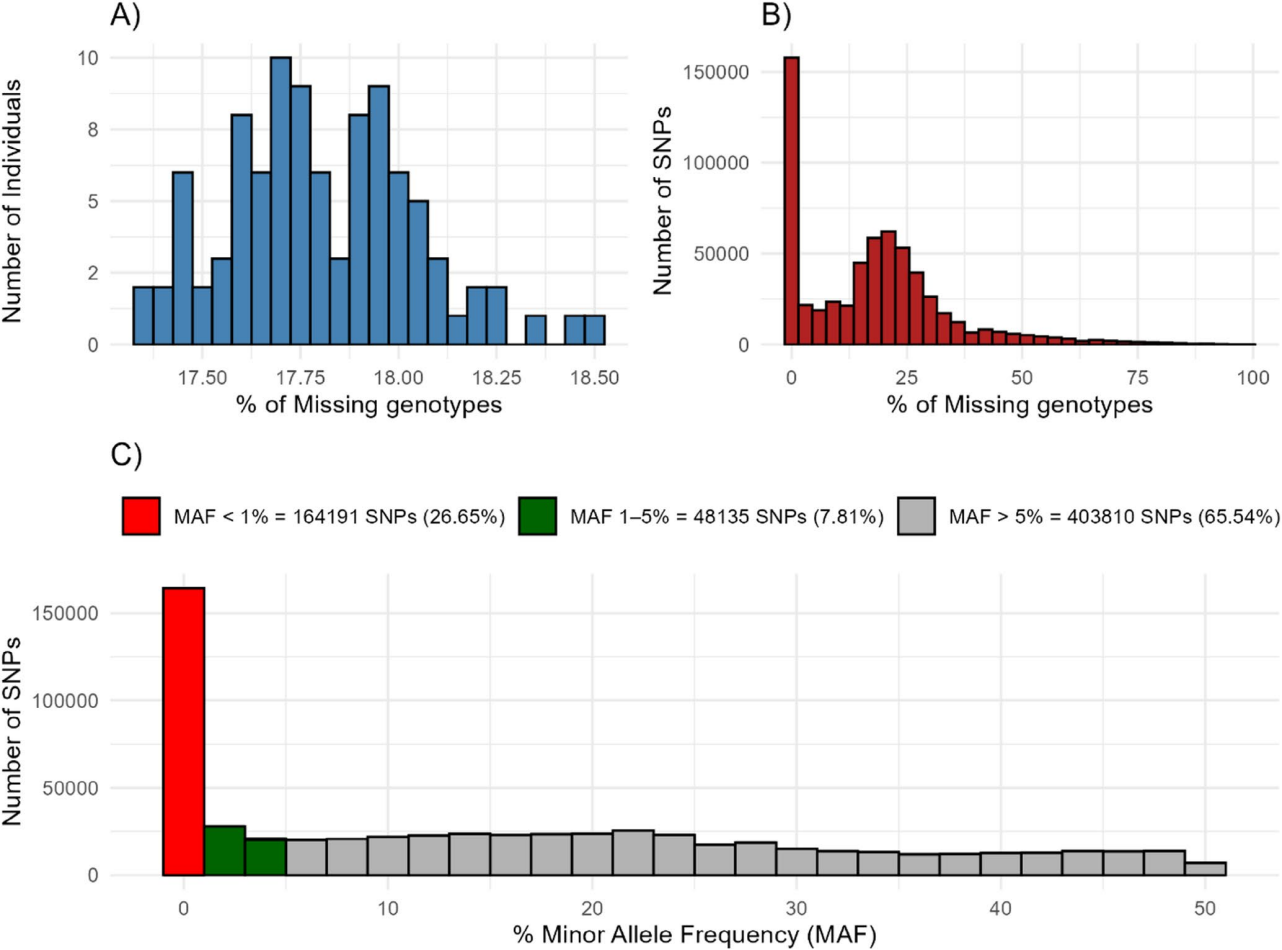
SNP chip statistics considering autosomal and sex chromosomes: (A) Missingness per individual; (B) Missingness per marker; (C) Minor Allele Frequency (MAF) distribution, coloured according to different MAF classes, with the corresponding number and percentage of SNPs. All metrics are expressed as percentages.

SNP genotyping was performed on 96 of the 104 captured individuals. A subset of samples was excluded due to low DNA quality and/or insufficient quantity. The total number of markers was 616,136 SNPs, and the average call rate for passing samples reported by the external lab was 81.05%. The Axiom Mouse HD was built on the *Mus musculus* genome assembly. The average call rate (81.05%) was lower than that achieved with SNP chips for livestock or companion animals, where a 90% threshold is easily reached. No individuals had missing values exceeding 20% (Figure 2A), with an overall average of missingness of 17% reported in individuals of both species (Table 1). A consistent number of monomorphic SNPs were identified (~150,000, Figure 2B), with a significant proportion of SNPs presenting 20%–25% of missing genotypes. To be sure to eliminate not only the monomorphic SNPs but also markers with a low minor allele frequency, a MAF threshold of 1% was tested, confirming that the removed variants satisfied the required criterion, as shown in Figure 2C.

**TABLE 1:** Missingness per individual of both species (expressed as %).

FID	Mean	SD	Maximum	Minimum
*A. flavicollis*	17.7	0.2	18.3	17.4
*A. sylvaticus*	17.9	0.2	18.5	17.3

Based on the aforementioned results, the threshold chosen for filtering missingness values was set at 20% for both genotypes and individuals. The MAF was set to 1%. Of the 616,136 SNPs, 594,475 were located on autosomes. Of the entire dataset ( *A. flavicollis* and *A. sylvaticus* ), 239,074 SNPs were removed due to missing genotype data, and a further 157,340 SNPs were removed due to the MAF threshold. The final entire dataset contained 198,061 markers and 96 samples. QC was carried out for each group using the same thresholds. The SNP‐based analyses independently resolved the individuals into two clearly distinct genetic clusters, which showed a high degree of concordance with the preliminary morphological species identification. The *A. flavicollis* dataset decreased from 594,475 autosomal markers to 175,708, with 243,862 variants removed due to—geno flag, and 174,905 variants removed due to the MAF threshold. All individuals were retained (*n* = 43). For the *A. sylvaticus* dataset, 168,685 variants and 53 individuals passed the filters and QC. A total of 248,442 SNPs were removed due to missing genotype data, and 177,348 variants were excluded due to low MAF values.

**FIGURE 3:**
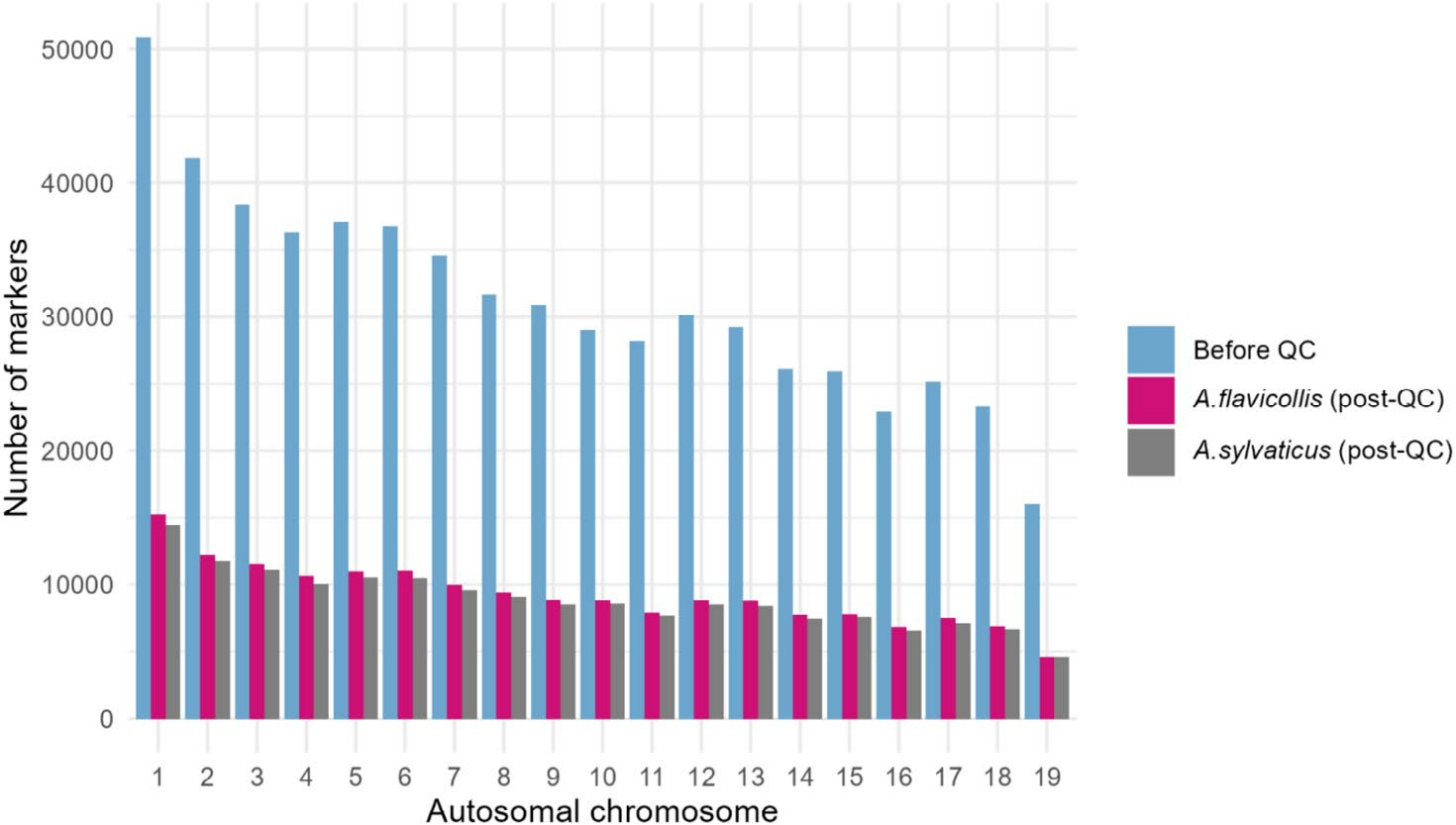
Number of SNPs among autosomal chromosomes divided by *A. flavicollis* and *A. sylvaticus* (SNPs are annotated and mapped according to the *Mus musculus* autosomes).

The distribution of SNPs among chromosomes (Figure 3) mapped according to the *Mus musculus* reference genome showed that the number of markers that passed the QC was similar in both species, with chromosome 1 having the highest number of variants. The number of SNPs decreased linearly up to the last 19th chromosome, with a value lower than 5000.

### 3.3 Genetic Diversity

Observed heterozygosity (*H*_o_), expected heterozygosity (*H*_e_), u*H*_e_ and inbreeding coefficient (*F*_IS_) were calculated for each species. The overall mean *H*_o_ for *A. flavicollis* was 0.335 ± 0.018, ranging from 0.302 to 0.382. Similar values were observed for *A. sylvaticus* (mean = 0.333 ± 0.015, minimum = 0.303, maximum = 0.367). Descriptive statistics for all metrics are reported in Table 2, separated by species and sampling site.

**TABLE 2:** Observed heterozygosity (*H*_o_), expected heterozygosity (*H*_e_), unbiased expected heterozygosity (u*H*_e_) and inbreeding coefficient (*F*_IS_) with the relative standard deviations (SD). Mean n.Ind per locus indicates the mean number of successfully genotyped individuals per locus used in the heterozygosity calculations.

	Sites	Mean sites n.Ind per locus	n.Loc	*H*_o_	*H*_o_ SD	*H*_e_	*H*_e_ SD	u*H*_e_	u*H*_e_ SD	*F*_IS_	*F*_IS_ SD
*A. flavicollis*	S1	6	175,708	0.343	0.311	0.247	0.187	0.268	0.203	−0.202	0.299
	S2	19	175,708	0.334	0.283	0.251	0.177	0.258	0.181	−0.197	0.263
	S3	4	175,707	0.340	0.330	0.237	0.195	0.268	0.220	−0.208	0.312
	S4	4	175,672	0.346	0.341	0.238	0.199	0.277	0.232	−0.195	0.320
	S5	2	172,856	0.350	0.413	0.208	0.224	0.289	0.311	−0.181	0.288
	S7	3	175,370	0.337	0.373	0.217	0.212	0.268	0.261	−0.211	0.324
*A. sylvaticus*	S3	4	168,678	0.345	0.311	0.250	0.189	0.283	0.213	−0.171	0.304
	S4	11	168,685	0.339	0.275	0.258	0.174	0.270	0.182	−0.182	0.261
	S5	16	168,685	0.333	0.269	0.256	0.172	0.264	0.178	−0.179	0.252
	S6	9	168,685	0.333	0.289	0.249	0.181	0.264	0.192	−0.187	0.278
	S7	6	168,685	0.335	0.308	0.242	0.188	0.264	0.205	−0.199	0.295

Expected heterozygosity values were consistently lower than *H*_o_ for both species, averaging around 0.24–0.25 across sites. u*H*_e_ remained remarkably stable, averaging around 0.26 across all populations of both species. *F*_IS_ estimates were consistently negative, indicating a slight excess of heterozygotes. The highest *F*_IS_ variability was observed in sites with smaller sample sizes (e.g., S5 and S7 in *A. flavicollis* ). However, the standard deviation of *F*_IS_ (*F*_IS_SD) equalled or exceeded the mean value in several populations, reflecting high locus‐to‐locus variability, especially in sites with small sample sizes. The overall magnitude and range of heterozygosity estimates were comparable between species, indicating similar levels of genetic diversity.

### 3.4 Population Structure

**FIGURE 4:**
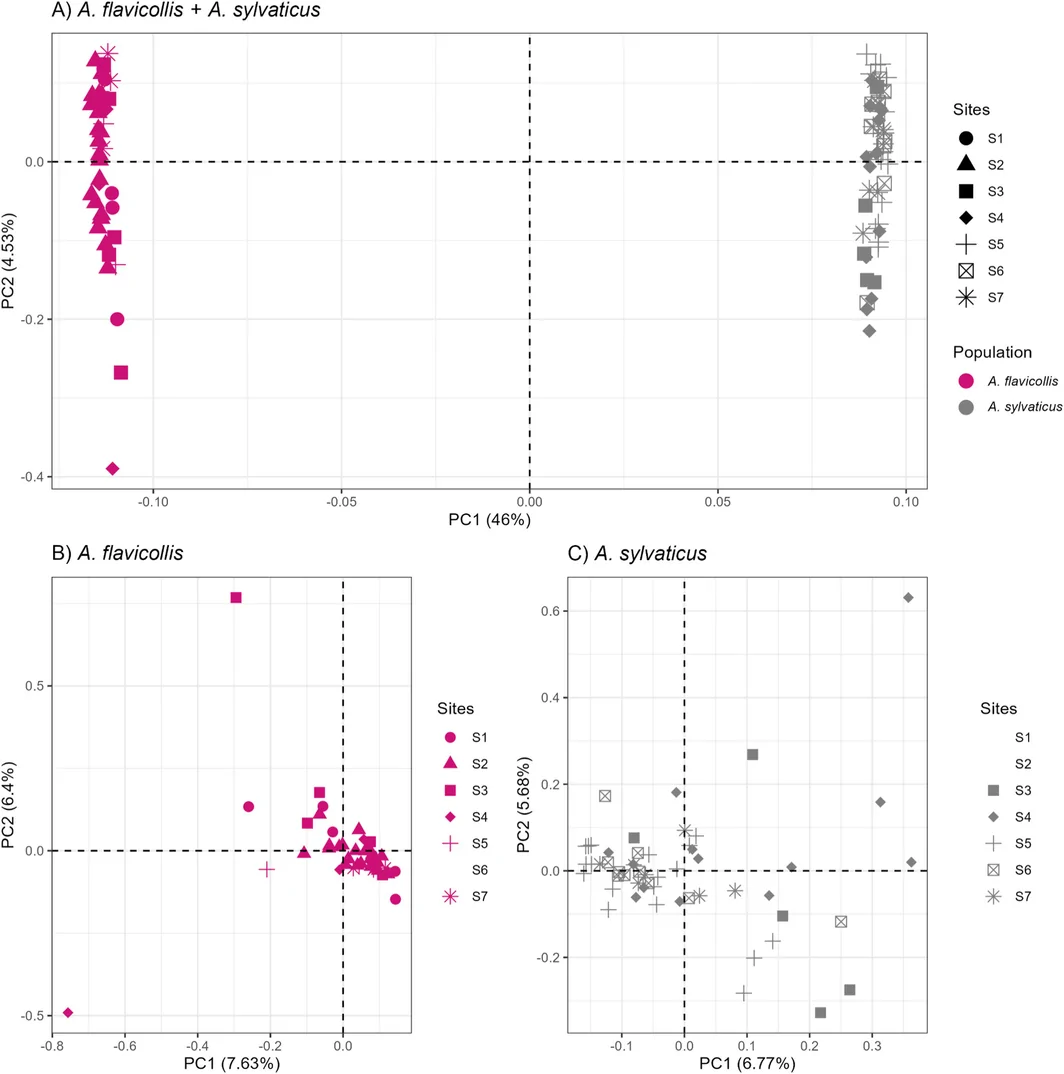
Principal Components Analyses on (A) the entire dataset, (B) on the *A. flavicollis* samples and (C) on the *A. sylvaticus* samples.

The applicability of the SNP chip to non‐conventional species was assessed by evaluating whether *A. flavicollis* and *A. sylvaticus* could be clearly differentiated into distinct genetic clusters. When both species were analysed together (Figure 4A), PC1 explained 46% of the total variance and clearly separated *A. flavicollis* and *A. sylvaticus* into two distinct clusters. In this case, PC1 did not discriminate among sites, as most of the genetic variation was explained by species‐level differences. When species were analysed separately (Figure 4B,C), the first two principal components explained < 15% of the total variance in both *A. flavicollis* and *A. sylvaticus* . Nevertheless, within‐species variability became more apparent, along with weak differentiation among sites. *Apodemus flavicollis* exhibited lower within‐species variability compared to *A. sylvaticus* , with individuals clustering more tightly and no clear separation among sites (Figure 4B). In contrast, *A. sylvaticus* showed higher genetic variability among individuals, with samples more widely dispersed (Figure 4C). However, even in this species, no clear genetic structuring among sites was observed.

**FIGURE 5:**
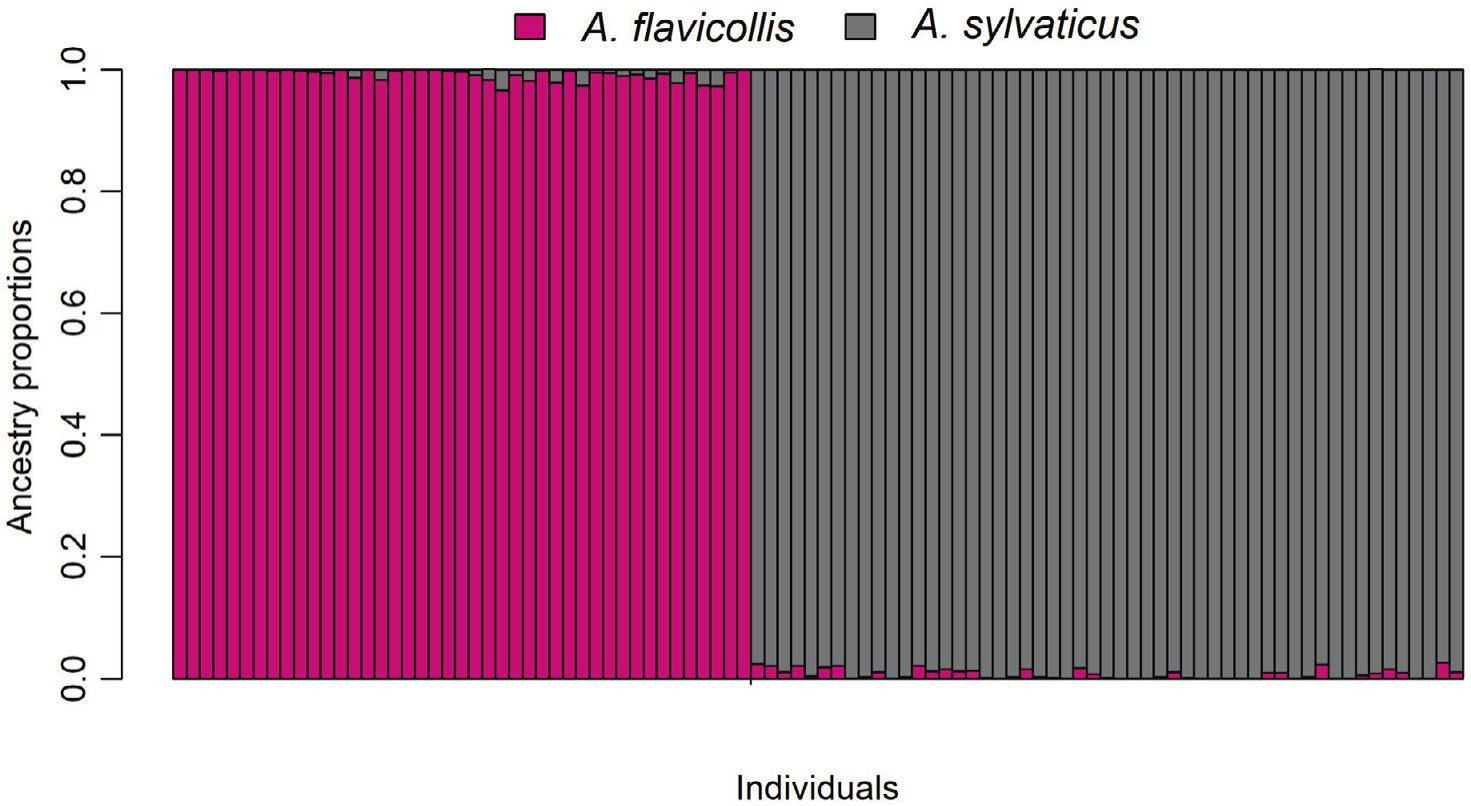
ADMIXTURE on the entire dataset (43 individuals of *A. flavicollis* and 53 individuals of *A. sylvaticus* ). Each vertical bar represents one individual, and the coloured segments indicate the estimated membership proportions (ancestry coefficients) assigned to each inferred genetic cluster.

To further support the PCA results, admixture analysis was performed. When the complete dataset was analysed (Figure 5), the best‐supported number of ancestral populations was *K* = 2 (Figure S1A), clearly separating *A. flavicollis* and *A. sylvaticus* into distinct genetic clusters.

**FIGURE 6:**
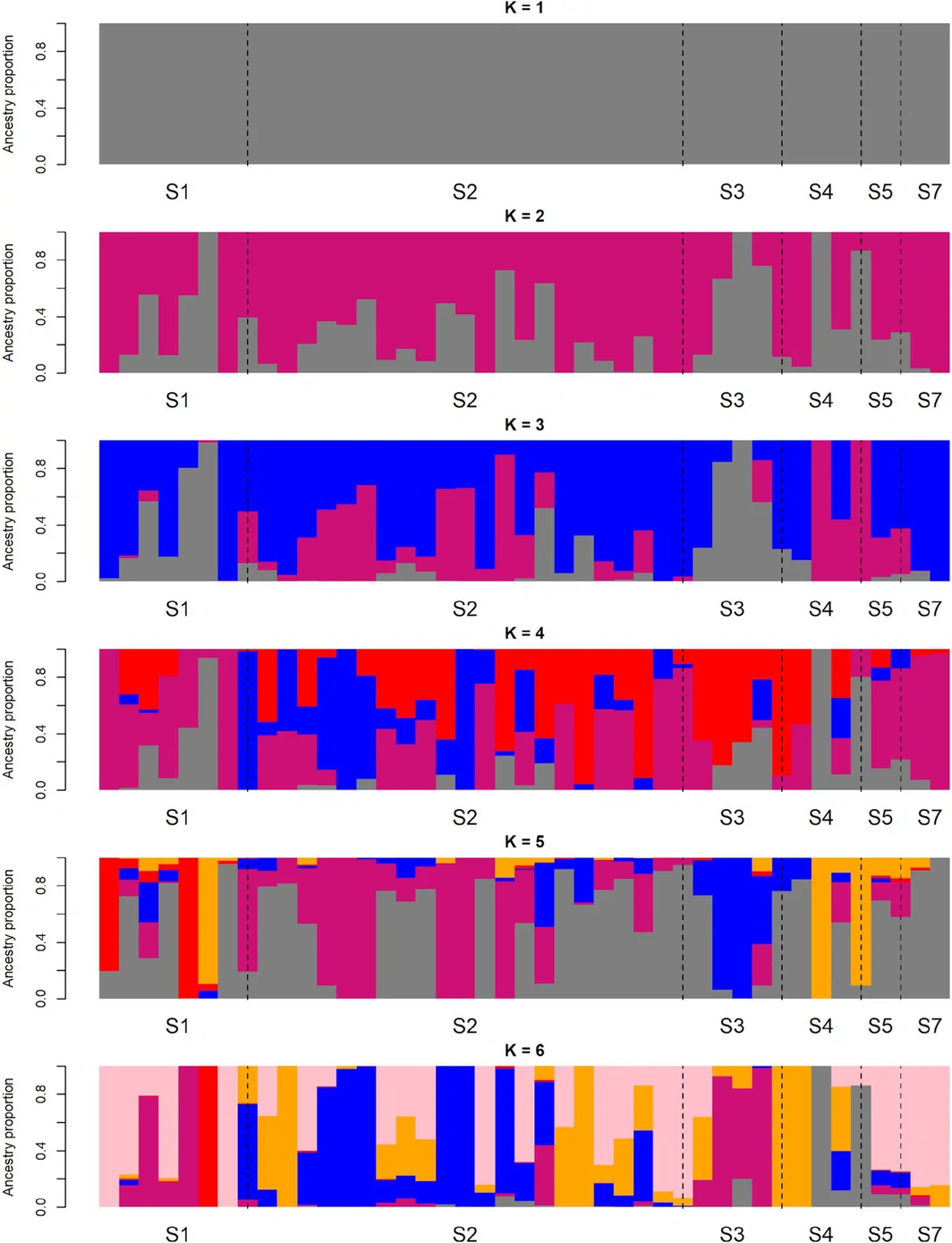
ADMIXTURE plot of *A. flavicollis* individuals assuming *K* = 1–6. Each vertical bar represents one individual, and the coloured segments indicate the estimated membership proportions (ancestry coefficients) assigned to each inferred genetic cluster. Individuals are grouped according to sampling site.

**FIGURE 7:**
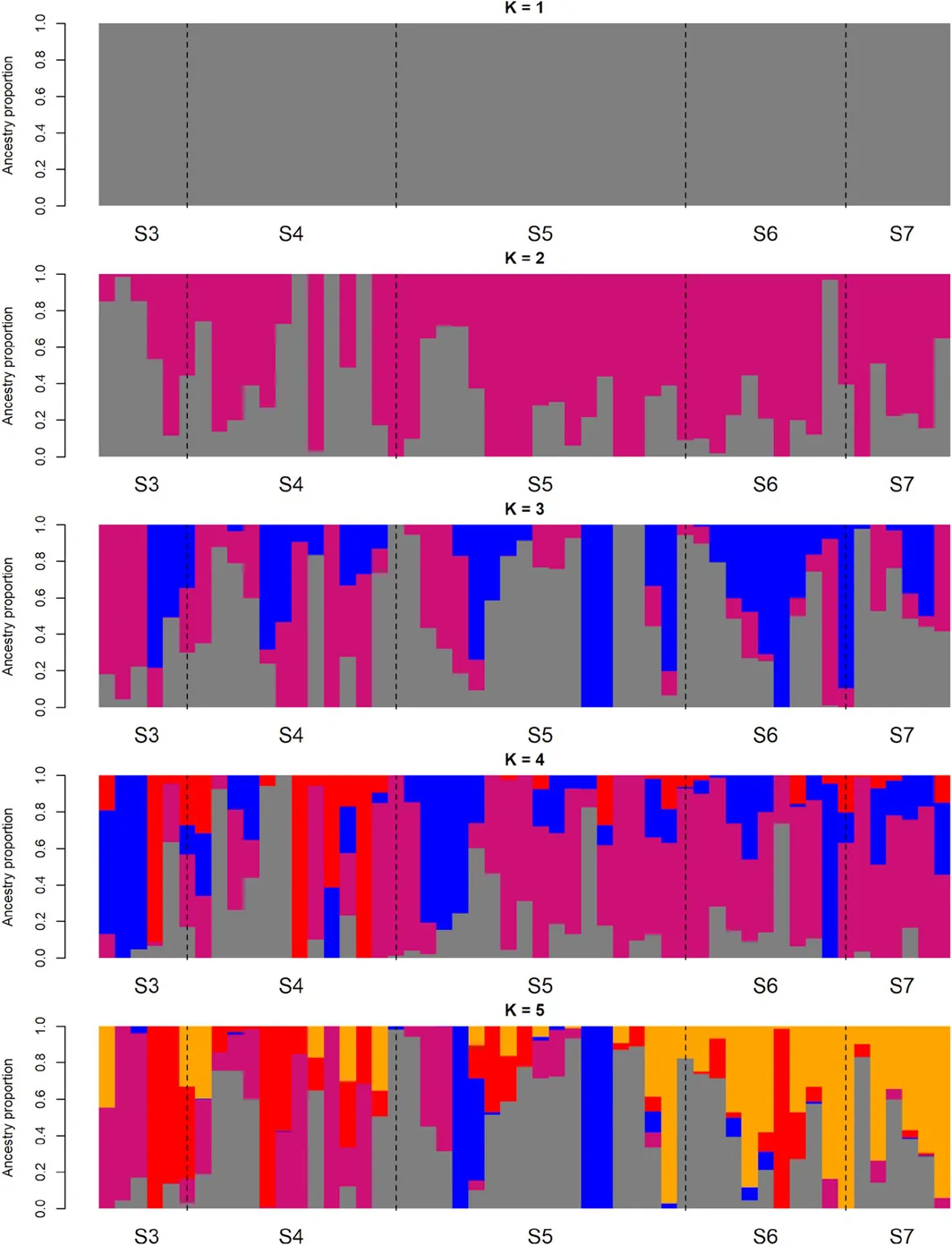
ADMIXTURE plot of *A. sylvaticus* individuals assuming *K* = 1–5. Each vertical bar represents one individual, and the coloured segments indicate the estimated membership proportions (ancestry coefficients) assigned to each inferred genetic cluster. Individuals are grouped according to sampling site.

Admixture analysis was repeated separately for each species, considering sampling sites as putative ancestral populations. For *A. flavicollis*, sites S1, S2, S3, S4, S5 and S7 were included (Figure 6), whereas for *A. sylvaticus* , sites S3, S4, S5, S6 and S7 were analysed (Figure 7).

In both species, the optimal number of ancestral populations was the *K* = 1 (Figure S1B,C), indicating that individuals within each species are best described as belonging to a single genetic cluster, with no clear subdivision among sampling sites. Although higher *K* values were explored, no consistent or well‐defined clustering patterns were observed. In *A. flavicollis* , sites S2, S4, S5 and S7 seemed to show partial similarity at *K* = 4, although these patterns were not stable across *K* values. In *A. sylvaticus* , S3–S4 and S6–S7 were observed at higher *K* values, including *K* = 5 (the maximum tested). However, these patterns were not consistent across *K* values. For instance, Site S5 appeared slightly differentiated only at *K* = 5, whereas at lower *K* values it showed greater similarity with S6 and S7. Overall, these results do not support clear genetic structuring among sites within either species.

**FIGURE 8:**
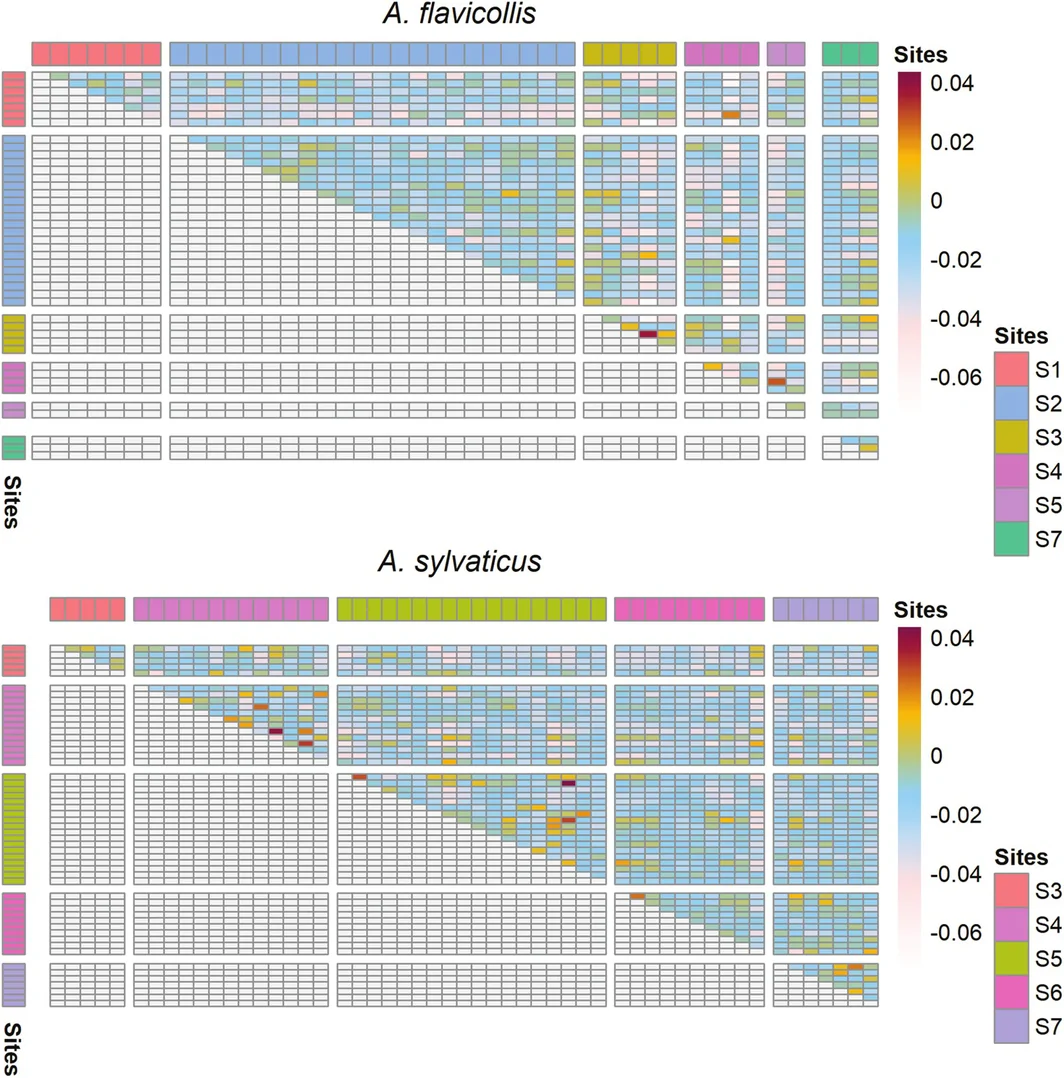
Heatmap of pairwise genomic relationship coefficients (derived from genome‐wide SNP data) within and among sampling sites in *A. flavicollis* and *A. sylvaticus*. Values close to zero indicate unrelated individuals, whereas increasingly positive values indicate higher levels of genetic relatedness between individuals.

Pairwise genomic relationship coefficients were calculated and are shown in Figure 8. Genetic relatedness was not restricted to within‐site comparisons, although, as expected, higher values were generally observed between individuals from the same site. In both species, relatedness was detected across all site combinations, and not particularly among geographically closer sites.

Interestingly, *A. flavicollis* showed a clearer distinction between highly related and unrelated individuals. In contrast, *A. sylvaticus* exhibited a more homogeneous distribution of relationship coefficients across individuals. This pattern may suggest a more uniform distribution of genetic similarity among sites, consistent with higher connectivity among sites in *A. sylvaticus* , which may reflect greater genetic exchange. This interpretation is further supported by the higher overall mean relatedness (in terms of genomic relationship coefficients) observed in *A. sylvaticus* (Table 3).

**TABLE 3:** Descriptive statistics within species and among sites of individual pairwise genomic relationship coefficients, with the total number of combinations (*n*_pairs).

Species		Mean	SD	*n*_pairs
*A. flavicollis*	Overall	−0.022	0.014	903
	S1	−0.019	0.012	21
	S2	−0.017	0.010	231
	S3	−0.013	0.030	10
	S4	−0.016	0.023	6
	S5	−0.002	—	1
	S7	−0.005	0.011	3
*A. sylvaticus*	Overall	−0.018	0.012	1378
	S3	−0.010	0.012	10
	S4	−0.013	0.017	78
	S5	−0.014	0.015	153
	S6	−0.013	0.011	45
	S7	−0.006	0.013	21

However, slightly higher mean relatedness values were observed at some sites: S5 and S7 in *A. flavicollis* (−0.002 and −0.005, respectively), and S7 in *A. sylvaticus* (−0.006). These patterns may be influenced by the smaller sample sizes of these sites (Table 3).

To investigate population subdivision among sampling sites, an analysis of molecular variance (AMOVA) was performed (Table 4). In both *A. flavicollis* and *A. sylvaticus* , the largest estimated variance component was attributed to variation within individuals (133.00% and 128.93%, respectively). The proportion of variance among populations (sampling sites) was very low but statistically significant in both species (0.56% in *A. flavicollis* and 0.44% in *A. sylvaticus* , *p* < 0.001). Variation among individuals within populations showed negative estimated variance components (−33.44% in *A. flavicollis* and −29.37% in *A. sylvaticus* ), indicating that genetic differentiation at this hierarchical level was effectively negligible. Negative variance component estimates may occur in AMOVA when the corresponding component of genetic variation is very small or absent and are generally interpreted as indicating no detectable variance at that hierarchical level rather than biologically meaningful negative variance (Excoffier et al. 1992, 2005; Grünwald and Hoheisel 2006). Overall, these results indicated very weak genetic structuring among sampling sites, with most of the genetic variation occurring within individuals.

**TABLE 4:** Population genetic diversity subdivision among sampling sites of *A. flavicollis* and *A. sylvaticus* .

Variations	*A. flavicollis*		*A. sylvaticus*	
	Sigma	%	Sigma	%
Among populations	124	0.56***	95	0.44***
Among individuals within populations	−7410	−33.44	−6399	−29.37
Within individuals	29,447	133.00	28,089	128.93
Total variations	22,161	100.00	21,786	100.00

*** *p* < 0.001.

The Mantel test revealed weak and non‐significant correlations between genetic and geographic distances in both species ( *A. flavicollis* : *r* = 0.178, *p* = 0.226; *A. sylvaticus* : *r* = −0.194, *p* = 0.73). These results indicate no clear pattern of isolation by distance in either species, suggesting that geographic distance is not a major driver of genetic differentiation in the study system.

## 4 Discussion

The primary aim of this study was to evaluate the performance of a non‐species‐specific SNP array for discriminating between *A. sylvaticus* and *A. flavicollis* and for genetically characterising these two closely related, morphologically similar non‐model species. Species of the genus *Apodemus* are often difficult to reliably identify in the field due to the strong overlap in external morphological traits (Bartolommei et al. 2016). Field identification based on morphology is particularly challenging when dealing with sibling species, where commonly used diagnostic characters show extensive overlap, making the correct assignment of individuals uncertain (Bartolommei et al. 2016). In southern regions such as continental Italy, including the Basilicata region where this study was conducted, *A. sylvaticus* and *A. flavicollis* show strong phenotypic convergence, especially in sympatric populations, further complicating species identification (Amori et al. 1986; Panzironi et al. 1994). Although estimates of genetic diversity obtained with the Axiom MouseHD panel should be interpreted cautiously, because the SNPs were originally selected from variation identified in a different species ( *Mus musculus* ), all individuals were genotyped using the same marker set and quality‐control procedures. Consequently, comparative analyses of population structure and connectivity are expected to be less sensitive to this source of bias. Nevertheless, because the true linkage architecture of *Apodemus* is not known with the same confidence as in *Mus musculus* , residual LD among physically linked markers cannot be entirely excluded. This represents an inherent limitation of using a cross‐species SNP array. Despite this limitation, the SNP‐based analyses independently resolved two distinct genetic clusters that showed a high degree of concordance with the preliminary species identification based on morphological and biometric characteristics, supporting the ability of the SNP array to discriminate between *A. sylvaticus* and *A. flavicollis* despite its original development for a different species. Beyond species assignment, the SNP array also proved informative for the genetic characterisation of both *A. sylvaticus* and *A. flavicollis* . As expected for a cross‐species genotyping approach, the overall call rate was lower than that typically reported for SNP arrays developed for target species or domestic animals (Sasaki et al. 2018), and a substantial proportion of markers were either monomorphic or had missing genotypes. This pattern is consistent with the application of SNP arrays outside their target species, where sequence divergence from the reference genome can reduce probe hybridisation efficiency and, consequently, SNP calling performance (Haynes and Latch 2012). Nevertheless, after quality filtering, a large number of informative autosomal SNPs were retained in both the combined and species‐specific datasets, while all individuals passed the filtering thresholds. This is a key result, indicating that despite the expected loss of loci in a cross‐species context, the array still provided sufficient genomic coverage for downstream analyses. The broadly similar number and chromosomal distribution of retained SNPs in the two species further support the consistency of the approach and suggest that comparable datasets can be generated across closely related non‐model taxa.

Building on the robustness of the SNP dataset, we further assessed patterns of genetic diversity within and among populations of both species. Overall, *A. flavicollis* and *A. sylvaticus* showed comparable levels of genetic diversity, with similar values of observed heterozygosity across sampling sites. In both species, observed heterozygosity consistently exceeded expected heterozygosity, resulting in negative *F*_IS_ values and indicating an excess of heterozygotes. The consistently negative *F*_IS_ values and the excess of observed over expected heterozygosity across populations deserve particular consideration. Although this pattern may be consistent with high connectivity and extensive mixing among individuals, technical effects associated with the cross‐species application of the SNP array cannot be excluded. In particular, ascertainment bias and the preferential retention of loci successfully genotyped in *Apodemus* may influence allele‐frequency‐dependent statistics, including heterozygosity and *F*_IS_. Therefore, these estimates should be interpreted cautiously and should not be considered solely as evidence of biological processes. Negative *F*_IS_ values were observed consistently across sites, although higher variability in *F*_IS_ estimates occurred in populations with smaller sample sizes, likely reflecting increased stochasticity and locus‐specific variation. Therefore, estimates from sites represented by only a few individuals should be interpreted with caution, as they are more susceptible to sampling effects. Nevertheless, the overall similarity in diversity levels among sites, significant differences in observed heterozygosity were detected between all pairwise comparisons. This suggests that, even in the absence of big differences in mean diversity estimates, the SNP dataset was sensitive enough to capture subtle genetic differences among populations. Such fine‐scale variation may reflect localised demographic processes or differences in connectivity across the landscape. Importantly, these results highlight the SNP array's ability to detect biologically meaningful variation in genetic diversity and inbreeding patterns, even in species characterised by relatively high dispersal potential and weak genetic structure (Jeffries et al. 2016).

Building on patterns of genetic diversity, we further investigated population structure and connectivity within each species. Consistent with the clear interspecific separation observed in the combined analyses, species‐specific analyses revealed a different pattern. When each species was analysed separately, the proportion of variance explained by the first principal components was low, and no clear clustering of individuals by sampling site emerged. Although some degree of individual variability was detectable, particularly in *A. sylvaticus* , overall genetic differentiation among sites was weak in both species. A few individuals occupied more extreme positions in the PCA. However, they passed all quality‐control filters and did not form distinct genetic clusters. Their distribution may reflect individual genetic variation or local demographic processes rather than discrete population subdivision, although further sampling would be required to clarify the underlying causes. Nevertheless, the overall pattern of weak genetic differentiation contrasts with what is often observed in species inhabiting human‐modified landscapes, where reduced genetic diversity within populations and increased differentiation among populations are common (Spielman et al. 2004; Willoughby et al. 2015). The lack of genetic structuring observed in *Apodemus* species in our study area suggests that these populations are not strongly affected by isolation among woodland fragments at the spatial scale investigated. This lack of structuring was further supported by additional analyses, all of which indicated the absence of clear population subdivision and no evidence of isolation by distance within the study area. However, given the small sample size, the non‐significant Mantel test result should be interpreted cautiously, as it does not necessarily indicate the absence of isolation by distance. Previous studies on *Apodemus* species have shown that habitat fragmentation can affect local distribution and abundance, with the species more frequently absent from woodlands located more than 2 km from other substantial woodland patches (Marsh et al. 2001). Taken together, our results indicate high levels of gene flow and functional connectivity among sampling sites in both species. The absence of strong genetic structure, even within a fragmented landscape, is consistent with the documented dispersal capacity of these species and suggests that dispersal may be sufficient to counteract the effects of genetic drift at the spatial scale considered. This is consistent with the ecological characteristics of *Apodemus* species, which are more habitat generalists than other small mammal rodents (Diaz et al. 1999). However, these findings suggest that ecological and genetic responses to fragmentation may occur at different spatial scales, as genetic structure can also be influenced by landscape features that restrict individual movement and gene flow, rather than by geographic distance alone (Gortat et al. 2010). Therefore, future studies spanning larger spatial scales and including more distant habitat fragments, while explicitly considering landscape barriers, will be essential to determine the conditions under which genetic differentiation emerges, as observed in other highly vagile small mammals (Ortiz et al. 2019). This would provide a more comprehensive understanding of the spatial scale of gene flow and connectivity in fragmented landscapes. Interestingly, differences between the two species emerged in terms of relatedness patterns. *Apodemus flavicollis* showed a clearer distinction between highly related and unrelated individuals, whereas *A. sylvaticus* exhibited a more homogeneous distribution of relatedness values across individuals and sites. This pattern may reflect higher connectivity and more widespread gene flow in *A. sylvaticus* , consistent with its greater ecological flexibility. Although both species occurred across most of the study area, their distribution was not completely overlapping, with *A. flavicollis* absent from S6 and *A. sylvaticus* absent from S1 and S2. While the limited spatial extent of the study and sample sizes do not allow firm ecological conclusions, this pattern is consistent with the known ecological differences between the two species. *Apodemus flavicollis* is more closely associated with woodland habitats, whereas *A. sylvaticus* shows greater ecological plasticity, being able to exploit a wider range of habitats and to move more effectively across heterogeneous and fragmented landscapes (Amori et al. 2008). However, some site‐specific signals of increased relatedness were observed, particularly in populations with smaller sample sizes, suggesting that local demographic effects or sampling limitations may influence these estimates.

Even in widespread and highly mobile species such as *Apodemus* species, maintaining functional connectivity is essential, as ongoing habitat fragmentation can affect metapopulation dynamics over time (Umbrello et al. 2022). Thus, knowledge of factors driving connectivity is crucial for supporting resilient populations of both threatened and non‐threatened species, characterised by relatively high dispersal and low genetic differentiation. The SNP array provided consistent and biologically meaningful insights into population connectivity, further supporting the use of high‐density SNP datasets as a powerful tool for investigating population genetic processes in non‐model species, where traditional markers may lack the resolution to detect subtle patterns of differentiation (Skey et al. 2023).

Together, these findings demonstrate that a single SNP‐based approach can simultaneously achieve accurate species discrimination and provide high‐resolution insights into population genetic processes. While ideally species‐specific SNP arrays would maximise genotyping efficiency and genomic coverage, their development is often economically unfeasible for most non‐model organisms, as genomic resources are typically driven by research priorities in human genetics and breeding applications (McMahon et al. 2014). In this context, our results highlight that cross‐species SNP arrays can represent a powerful and cost‐effective alternative, enabling both taxonomic identification and population genetic analyses within a unified framework, even in closely related wild species.

## Author Contributions

**Maria Chiara Fabbri:** conceptualization (lead), data curation (lead), formal analysis (lead), investigation (equal), visualization (lead), writing – original draft (lead), writing – review and editing (equal). **Matilde Martini:** conceptualization (lead), data curation (equal), investigation (lead), visualization (equal), writing – original draft (equal), writing – review and editing (lead). **Giovanna Donati:** data curation (equal), investigation (equal), writing – review and editing (equal). **Giuseppe Coiro:** investigation (equal), writing – review and editing (supporting). **Giuseppe Luzzi:** resources (lead), writing – review and editing (supporting). **Luciano Ferraro:** writing – review and editing (supporting). **Donata Coppola:** writing – review and editing (supporting). **Riccardo Bozzi:** conceptualization (equal), funding acquisition (lead), project administration (lead), supervision (lead), validation (lead), writing – review and editing (lead).

## Funding

This work was funded by the FESR FSE+ Basilicata 2021–2027 Regional Programme. Authorisation for field sampling was granted by the Park Authority as part of the project (CCI no. 2021IT16FFPR004).

## Conflicts of Interest

The authors declare no conflicts of interest.

## Supporting information


**Figure S1:** Cross‐entropy values for the full dataset (A) and for the reduced datasets (B: *A. flavicollis* ; C: *A. sylvaticus* ).

## Data Availability

All genomic data are archived on Zenodo: https://zenodo.org/records/19858711?preview=1&token=eyJhbGciOiJIUzUxMiJ9.eyJpZCI6ImFlMzllZjMwLTEzY2YtNDZmOC1iNGE0LWQyYjQ4NDBmZTQ4OCIsImRhdGEiOnt9LCJyYW5kb20iOiIyNTdhYjhkMjQ4NjQzYmYyMzNlMmUzZmY4YzRkMDUyMSJ9.viSz2awUCvHSWsIZ_U94iTNPsnTcu8SCjUZeHnXxwY68O5HNQ_1apFYH‐zd0tUumhzyqm10Xz7bnryR253J58A.
