# Supplementary material for: High‐Density SNP Genotyping Reveals High Population Connectivity and Limited Spatial Genetic Structure in Apodemus flavicollis and Apodemus sylvaticus
Source: Ecol Evol. 2026 Sep 8;16(9):e74345. doi: 10.1002/ece3.74345 (PMC13553552; doi:10.1002/ece3.74345)
Supplement: Supplementary file 1 [file ECE3-16-e74345-s001.pdf]

Supplementary Figure 1: Cross-entropy values for the full dataset (A) and for the reduced datasets (B: *A. flavicollis*; C: *A. sylvaticus*).

A)

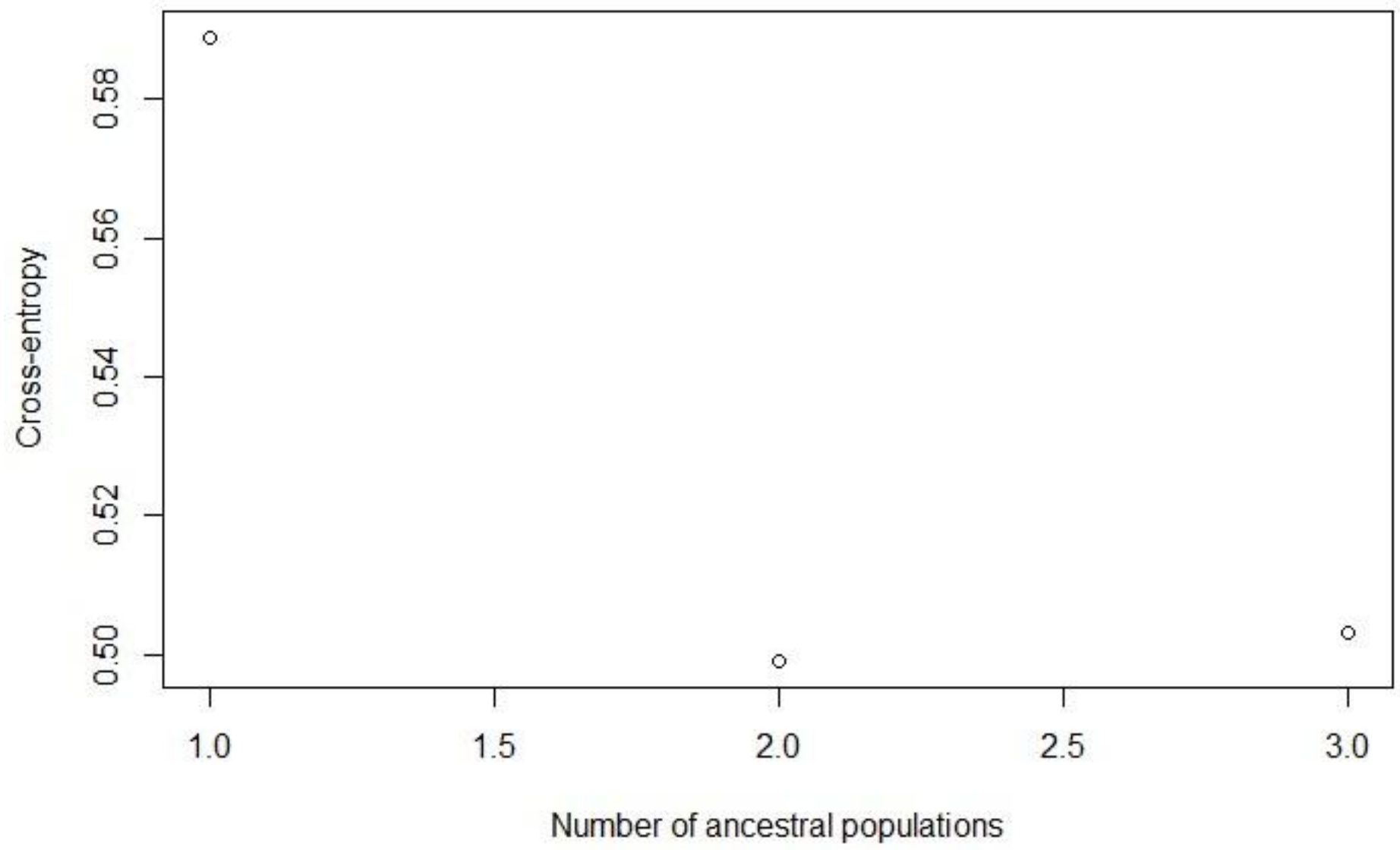

B)

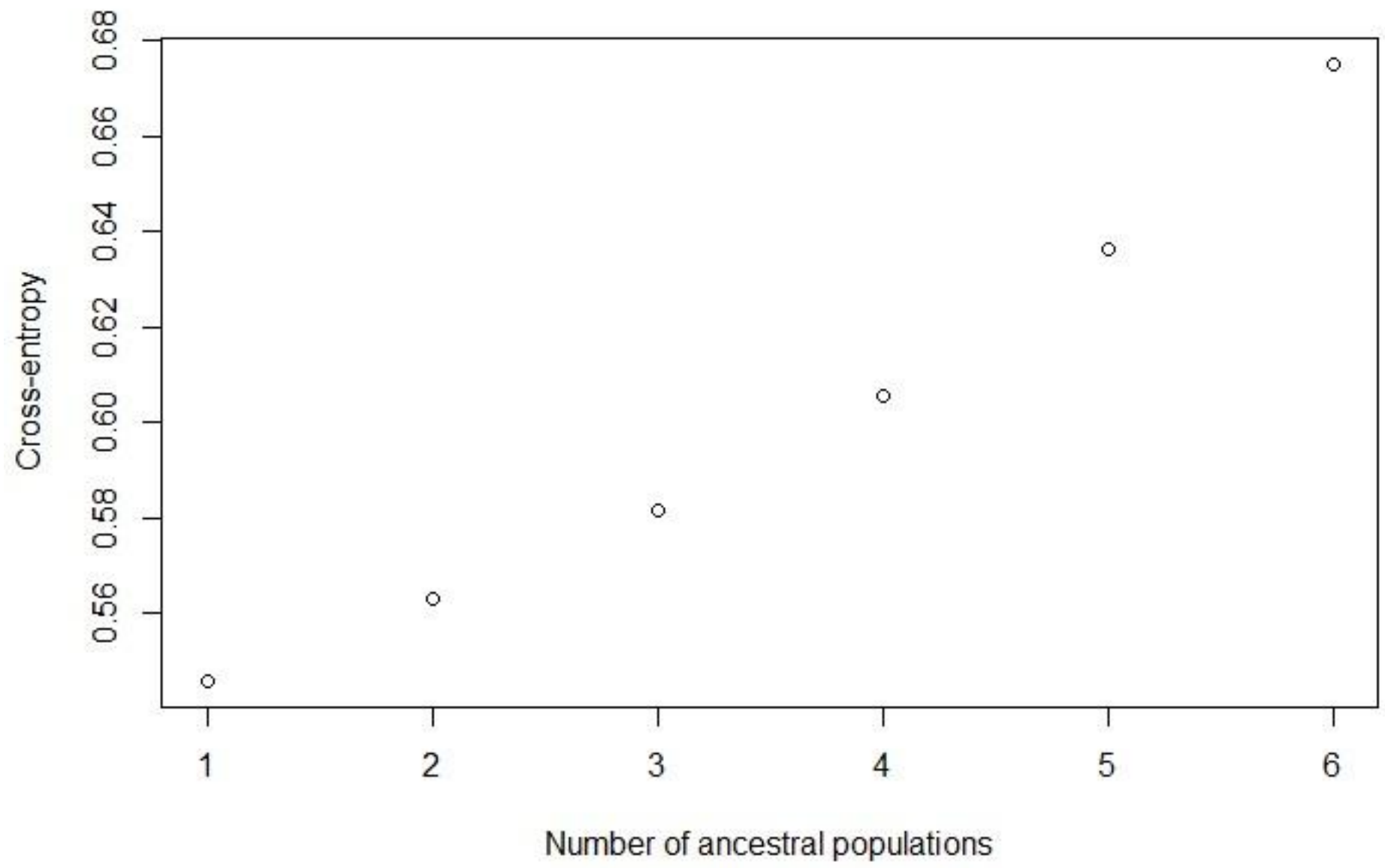

C)

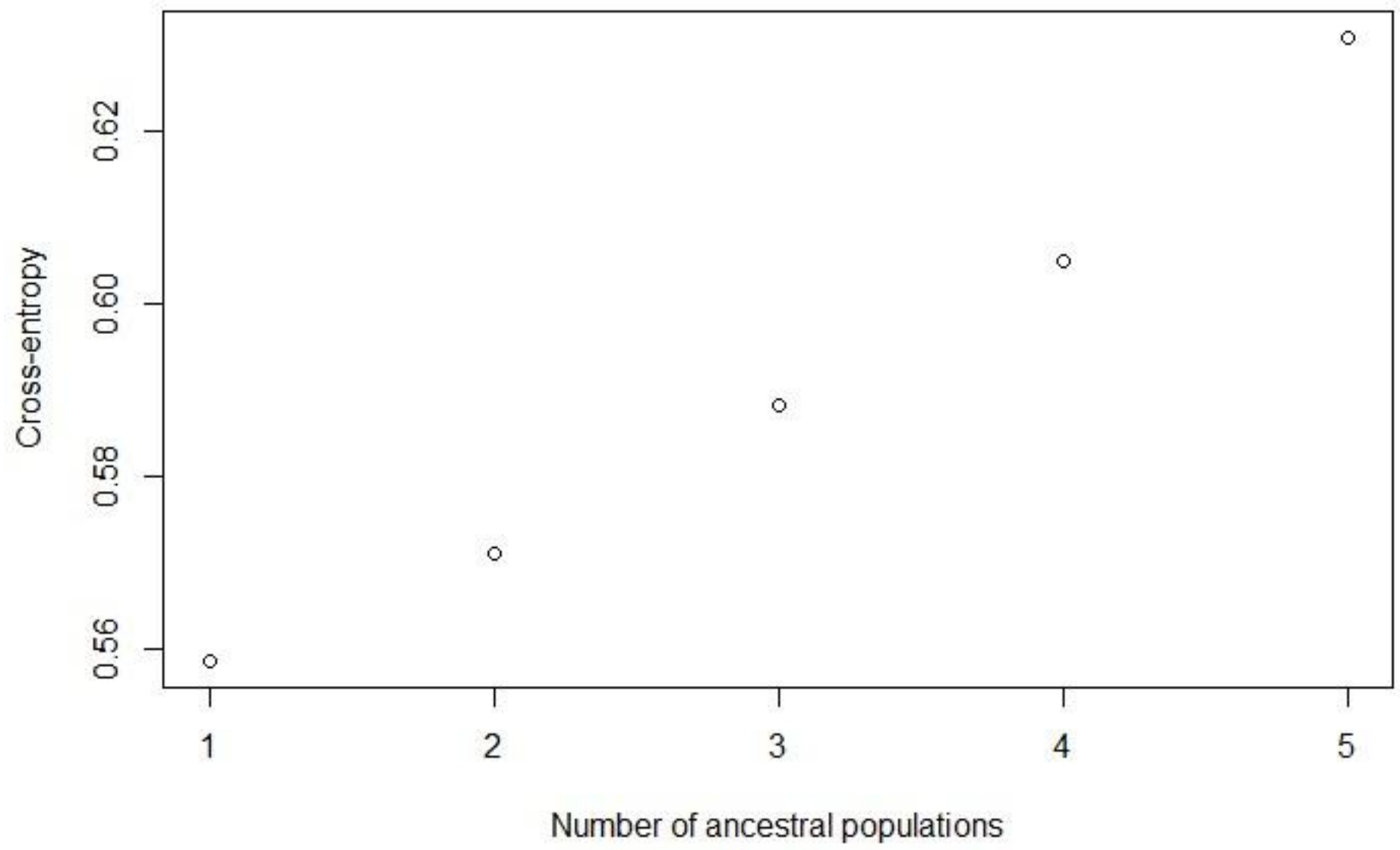

Cross-entropy
0.56
0.58
0.60
0.62
1
2
3
4
5
Number of ancestral populations
